# Molecular Targets and Associated Potential Pathways of Danlu Capsules in Hyperplasia of Mammary Glands Based on Systems Pharmacology

**DOI:** 10.1155/2017/1930598

**Published:** 2017-05-23

**Authors:** Jihan Huang, Haitao Tang, Sumin Cao, Yingchun He, Yibin Feng, Kun Wang, Qingshan Zheng

**Affiliations:** ^1^Center for Drug Clinical Research, Shanghai University of Traditional Chinese Medicine, Shanghai 201203, China; ^2^School of Chinese Medicine, Li Ka Shing Faculty of Medicine, The University of Hong Kong, Pok Fu Lam, Hong Kong; ^3^Suzhong Pharmaceutical Group Co., Ltd., Taizhou 225500, China

## Abstract

Hyperplasia of mammary glands (HMG) is common in middle-aged women. Danlu capsules (DLCs) can effectively relieve pain and improve clinical symptoms and are safe for treating HMG. However, the active substances in DLCs and the molecular mechanisms of DLCs in HMG remain unclear. This study identified the bioactive compounds and delineated the molecular targets and potential pathways of DLCs by using a systems pharmacology approach. The candidate compounds were retrieved from the traditional Chinese medicine systems pharmacology (TCMSP) database and analysis platform. Each candidate's druggability was analyzed according to its oral bioavailability and drug-likeness indices. The candidate proteins and genes were extracted in the TCMSP and UniProt Knowledgebase, respectively. The potential pathways associated with the genes were identified by performing gene enrichment analysis with DAVID Bioinformatics Resources 6.7. A total of 603 compounds were obtained from DLCs, and 39 compounds and 66 targets associated with HMG were obtained. Gene enrichment analysis yielded 10 significant pathways with 34 targets. The integrated HMG pathway revealed that DLCs probably act in patients with HMG through multiple mechanisms of anti-inflammation, analgesic effects, and hormonal regulation. This study provides novel insights into the mechanisms of DLCs in HMG, from the molecular level to the pathway level.

## 1. Introduction

Hyperplasia of mammary glands (HMG), a common disease in middle-aged women, is a precancerous lesion of mammary glands. The morbidity rate of HMG is increasing annually and presents a younger trend [1]. According to theories from Western medicine, HMG is caused by the thymus-hypothalamus-pituitary-gonadal axis in normal immune processes and autoimmunity [2]. In particular, estrogen is produced abnormally and continuously stimulates the breast tissues, leading to the excessive proliferation of normal breast tissues. HMG is classified in the “Rupi” category in traditional Chinese medicine (TCM). According to TCM theory, HMG is a liver and kidney deficiency syndrome and a Chong–Ren dysfunction syndrome. Although these two syndromes are theoretically distinct, their main clinical manifestations are hyperplasia of mammary glands (HMG) and breast pain. HMG can be confirmed by mammography check-up or color ultrasonography [3]. Western medicine recommends hormone replacement therapy, which is associated with many adverse effects [4]. TCM has been widely used in China in clinical treatments for 2,000 years. Studies have reported the effectiveness of TCM in treating HMG [5, 6].

Danlu capsules (DLCs) are approved by the China Food and Drug Administration (Approval number Z20150004) and are widely available in China. Danlu capsules were called Kerutong capsules before being approved. The DLCs are composed of eight Chinese herbs, namely, Fructus Cnidii (CF, She Chuang Zi), Cortex Moutan (CM, Mu Dan Pi), Radix Paeoniae Rubra (RPR, Chi Shao), Radix Curcumae (RC, Yu Jin), Thallus Laminariae (TL, Kun Bu), Radix Polygonum Multiflorum Preparata (RPMP, He Shou Wu), Cornu Cervi (CC, Lu Jiao), and* Crassostrea gigas* (CG, Mu Li). The DLCs exert many effects, such as nourishing the liver and kidneys, regulating Chong and Ren channels, regulating qi and activating blood circulation, dissipating phlegm and resolving masses, eliminating stagnation, and alleviating pain.

Preclinical pharmacological studies have reported that DLCs have analgesic effects and have obvious anti-inflammatory effects for acute and chronic inflammations. Pathology studies showed that DLCs can inhibit HMG [7]. Furthermore, clinical trials have proven that DLCs effectively relieve pain, improve clinical symptoms, and are safe for treating HMG [8, 9]. Phase II and III clinical trials of DLCs also verified its clinical effect in patients with HMG and did not find obvious adverse effect. DLC has been approved by China Food and Drug Administration (Approval number Z20150004) and is widely used for treating dysmenorrhea in China. After confirming HMG, Danlu capsules (DLCs) treatment group is the HMG patients having breast pain simultaneously. However, the active compounds in DLCs and specific molecular mechanisms of DLCs in treating HMG remain unclear.

ATCM formula is a complex system composed of multiple components and targets as well as synergistic interactions among the components [10]. Because of its complex chemical composition, studying the role of the mixture in the body is difficult. Furthermore, this complexity makes it difficult to conduct a comprehensive study of TCM, whereas systems pharmacology [11] and network pharmacology [12] can help explore the molecular mechanisms of TCM formulae. The herbal compounds in orally administered TCM formulae must first overcome the barriers posed by absorption, distribution, metabolism, and excretion (ADME) processes, and only the molecules that pass through those barriers may be active [13]. These molecules bind to the targets in the body and thus achieve efficacy by interacting with the human body at the network and overall organ levels.

Therefore, in this study, on the basis of the ADME processes, we identified the active molecules in DLCs that cross the physiological barriers and predicted the network targets of these active substances. We also determined the overall effects of DLCs on the body and DLCs mechanisms of action, which may provide a basis for a more comprehensive understanding of the mechanisms of action of DLCs in the treatment of HMG.

## 2. Materials and Methods

### 2.1. Identification of Candidate Compounds

All compounds of the eight herbs in DLCs were retrieved from the TCM systems pharmacology (TCMSP) database and analysis platform. The TCMSP includes information on all 500 TCM formulae registered in the Chinese Pharmacopoeia (2010 edition), with a total of 30,069 ingredients retrieved through literature mining and database integration [13].

### 2.2. Screening for Active Compounds

Oral bioavailability (OB) [14] is one of the most important pharmacokinetic parameters in ADME processes. High OB is often a key indicator to determine the drug-likeness (DL) index of bioactive molecules. Most compounds in TCM formulae cannot reach the protein target sites in particular cells because they lack appropriate pharmacological properties, particularly OB. In the present study, the molecules with OB ≥ 30% were considered to have high OB.

The DL index is a qualitative concept used in drug design for estimating the druggability of a substance [15]. In the early stages of drug development, the evaluation of DL facilitates screening for excellent compounds and increases the hit rate of the drug candidates. Therefore, in this study, the DL indexes of molecules in DLCs were assessed using the Tanimoto coefficient [16], calculated using the following formula:

(1) $$\begin{array}{c} T\left(\;X,Y\;\right)=\frac{x*y}{x^{2}+y^{2}-x*y}, \end{array}$$

where *x* is the molecular descriptor of DLCs on the basis of Dragon software (http://www.talete.mi.it/products/dragon_description.htm) and *y* is the average descriptor of all drugs in the DrugBank database. The average DL index of all drugs in this database is 0.18. Thus, in this study, the active molecules were defined as those with DL indices of ≥0.18. The compounds with a DL index of >0.18 were considered to exhibit relatively favorable DL and were selected as candidate molecules for further analyses.

### 2.3. Identification of Drug Targets

The protein targets of the compounds were retrieved from the TCMSP (http://lsp.nwu.edu.cn/tcmsp.php). Then the gene names were extracted from UniProtKB (http://www.uniprot.org) and the targets were mapped to the therapeutic target database (TTD, http://bidd.nus.edu.sg/group/cjttd/TTD_HOME.asp), Comparative Toxicogenomics Database (CTD, http://ctdbase.org/), Pharmacogenomics Knowledge Base (PharmGKB, https://www.pharmgkb.org/), and Kyoto Encyclopedia of Genes and Genomes (KEGG, http://www.kegg.jp/) to obtain their corresponding diseases and associated targets.

### 2.4. Gene Ontology Enrichment Analysis for Targets

The gene ontology (GO) biological process (BP) was analyzed to further validate whether the potential targets are indeed a match for HMG. The GO enrichment analysis was performed using the functional annotation tool of DAVID Bioinformatics Resources 6.7 (https://david-d.ncifcrf.gov/). The terms from the GOBP indicates gene functions. The terms with Expression Analysis Systematic Explorer scores of ≤0.05 were selected for functional annotation clustering. Analysis for determining the false discovery rate (FDR), a multiple hypothesis testing error, yielded a *p* value of ≤0.05, which was used as the significance cutoff in our study.

### 2.5. Network Construction and Analysis

To comprehensively understand the molecular mechanisms of DLCs, the compound-target and target-pathway networks were constructed using Cytoscape 3.3.0 [17]. The compound-target network was constructed by linking the active compounds with their potential targets. The target-pathway network was generated by linking the signaling pathway with a target if the target exists in the signaling pathway. The significant pathways were identified by performing enrichment analysis of the proteins by using DAVID Bioinformatics Resources 6.7. In this bilateral network, nodes represented the compounds, targets, or signaling pathways, and edges represented the compound-target or target-pathway interactions.

## 3. Results

### 3.1. Identification of Active Compounds in DLCs

From the TCMSP, a total of 603 compounds were retrieved, namely, 114 in FC, 55 in CM, 119 in RPR, 222 in RC, 48 in TL, 28 in RPMP, 8 in CC, and 9 in CG. The eight herbs shared 81 compounds (Supplement 1, in Supplementary Material available online at https://doi.org/10.1155/2017/1930598). Of the 114 compounds in FC, 69 (64.2%) satisfied the criteria of OB ≥ 30% and 19 (24.5%) satisfied the criteria of OB ≥ 30% and DL ≥ 0.15. Of the 55 compounds in CM, 26 (28.3%) satisfied the criteria of OB ≥ 30% and 11 (17.4%) satisfied the criteria of OB ≥ 30% and DL ≥ 0.18. Of the 119 compounds in RPR, 58 (48.9%) satisfied the criteria of OB ≥ 30% and 29 (15.3%) satisfied the criteria of OB ≥ 30% and DL ≥ 0.15. Of the 222 compounds in RC, 142 (64.0%) satisfied the criteria of OB ≥ 30% and 15 (4.8%) satisfied the criteria of OB ≥ 30% and DL ≥ 0.15. Of the 48 compounds in TL, 27 (56.3%) satisfied the criteria of OB ≥ 30% and 7 (14.6%) satisfied the criteria of OB ≥ 30% and DL ≥ 0.15. Of the 28 compounds in RPMP, 11 (39.3%) satisfied the criteria of OB ≥ 30% and 8 (28.6%) satisfied the criteria of OB ≥ 30% and DL ≥ 0.15. Among the 603 compounds, a total of 89 satisfied all the aforementioned conditions (Table 1) and 77 compounds were finally obtained after excluding the duplicates. However, CC and CG were mainly composed of amino acids, inorganic mineral elements, and proteins and were not included in this study [18, 19].

### 3.2. Targets Identification of DLCs

In total, 51 of the 77 compounds in DLCs were associated with 742 target proteins. Supplement 2 presents detailed information on the obtained target proteins. After eliminating the overlapping proteins, 128 associated proteins were obtained. Subsequently, the targets were mapped to the TTD, CTD, PharmGKB, and KEGG. Finally, 66 targets associated with HMG were reserved (Table 2), and 39 compounds were obtained after removal of 38 candidate compounds without any relevant targets. Supplement 3 presents detailed information on the obtained active compounds.

### 3.3. GO Enrichment Analysis for Targets

**Figure 1:**
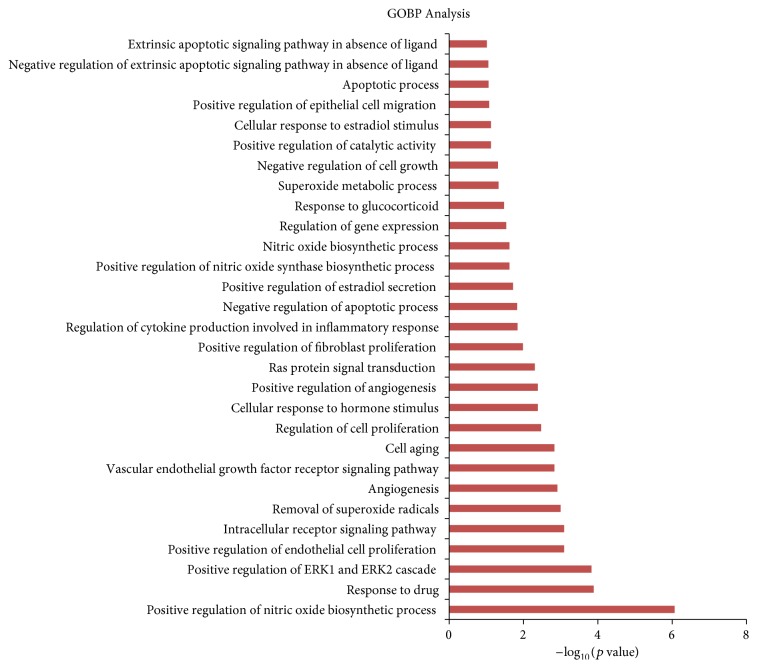
GO analysis of therapy targets. The *x*-axis represents the enrichment scores of these terms (*p* ≤ 0.05), and the *y*-axis represents significantly enriched BP categories in GO relative to the targets.

A GO enrichment analysis was performed using DAVID Bioinformatics Resources 6.7 to further validate whether the potential targets matched HMG.  Figure 1 lists the 30 most significantly enriched GOBP terms (*p* ≤ 0.05). Supplement 4 shows *p* values and FDR. The results revealed that numerous targets are involved in inflammatory reactions, estradiol secretion, and endothelial cell proliferation. Notably, the regulation of cytokine production is involved in inflammatory responses, regulation of cell proliferation, and cellular responses to estradiol stimulus, which are closely related to the pathogenesis of HMG.

### 3.4. Disease-Compound-Target Network and Analysis

**Figure 2:**
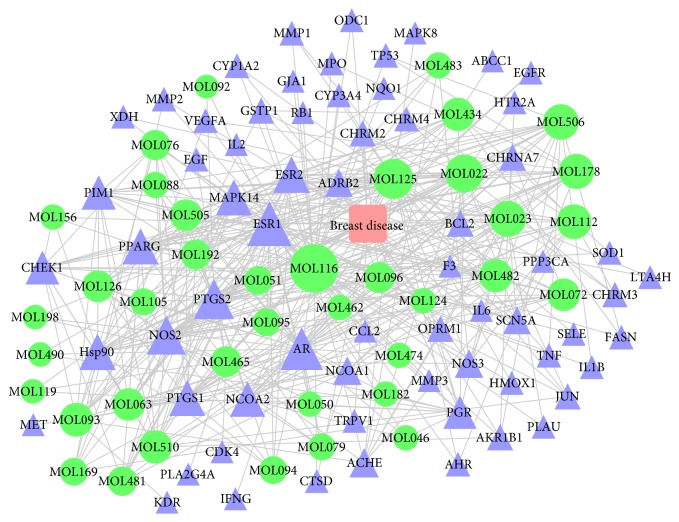
Disease-compound-target network for DLCs. The red, green, and blue nodes represent the disease, compounds, and targets, respectively. The edges represent the interactions among them and nodes sizes are proportional to their degree.

In total, 39 of the 77 compounds in DLCs were associated with 432 target proteins. After eliminating the overlapping proteins, 66 targets associated with HMG were obtained. Supplement 2 provides detailed information on the obtained target proteins. A disease-compound-target network was constructed on the basis of the 39 bioactive compounds and their targets. As shown in Figure 2, the network is composed of 106 nodes (1 disease, 39 bioactive compounds, and 66 targets). The red, green, and blue nodes represent the disease, compounds, and targets, respectively; the edges represent the interactions among them and nodes sizes are proportional to their degree. Subsequently, network analysis was performed by evaluating the centralization and heterogeneity. The resulting centralization and heterogeneity of the network are 0.384 and 1.067, respectively, indicating that some nodes are more concentrated in the network than others. In brief, the compound-target space is biased toward certain compounds and targets. Therefore, this network includes some compounds with multiple targets, particularly the high-degree compounds MOL116 (quercetin, degree = 48), MOL125 (kaempferol, degree = 29), MOL022 (beta-sitosterol, degree = 24), MOL023 (stigmasterol, degree = 20), MOL506 (aloe-emodin, degree = 19), MOL178 (baicalein, degree = 18), and MOL112 (o-isovalerylcolumbianetin, degree = 17).

### 3.5. Target-Pathway Network and Analysis

**Figure 3:**
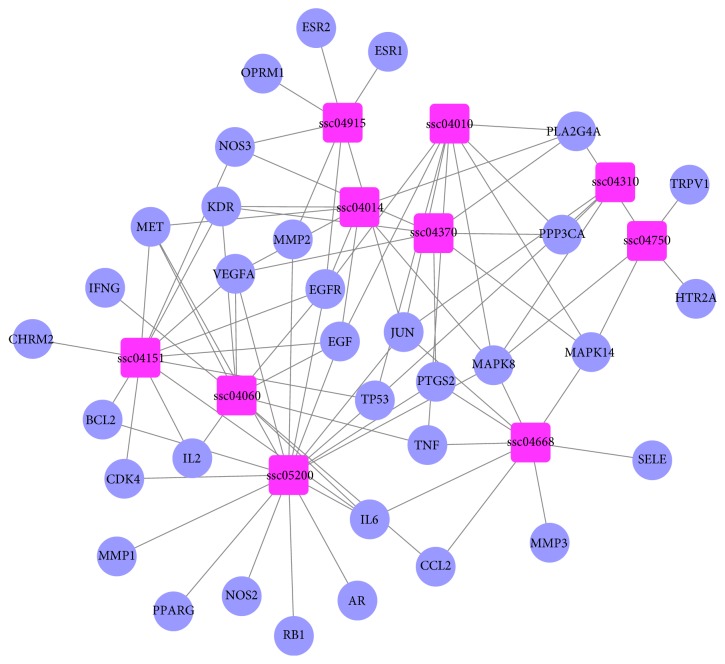
Target-pathway network for HMG. The pink and blue nodes represent the pathway and targets, respectively, and the edges represent the interactions among them.

The KEGG pathway enrichment analysis was performed using the functional annotation tool of DAVID Bioinformatics Resources 6.7. In total, 10 pathways were significantly associated with the input set of the targets (Supplement 3). To further elucidate the molecular mechanisms, we constructed a target-pathway network on the basis of all targets and their corresponding significant signaling pathways (Figure 3). As shown in Figure 3, this network is composed of 44 nodes (10 pathways and 34 proteins) and 97 interactions.

### 3.6. HMG Pathway and Therapeutic Modules

**Figure 4:**
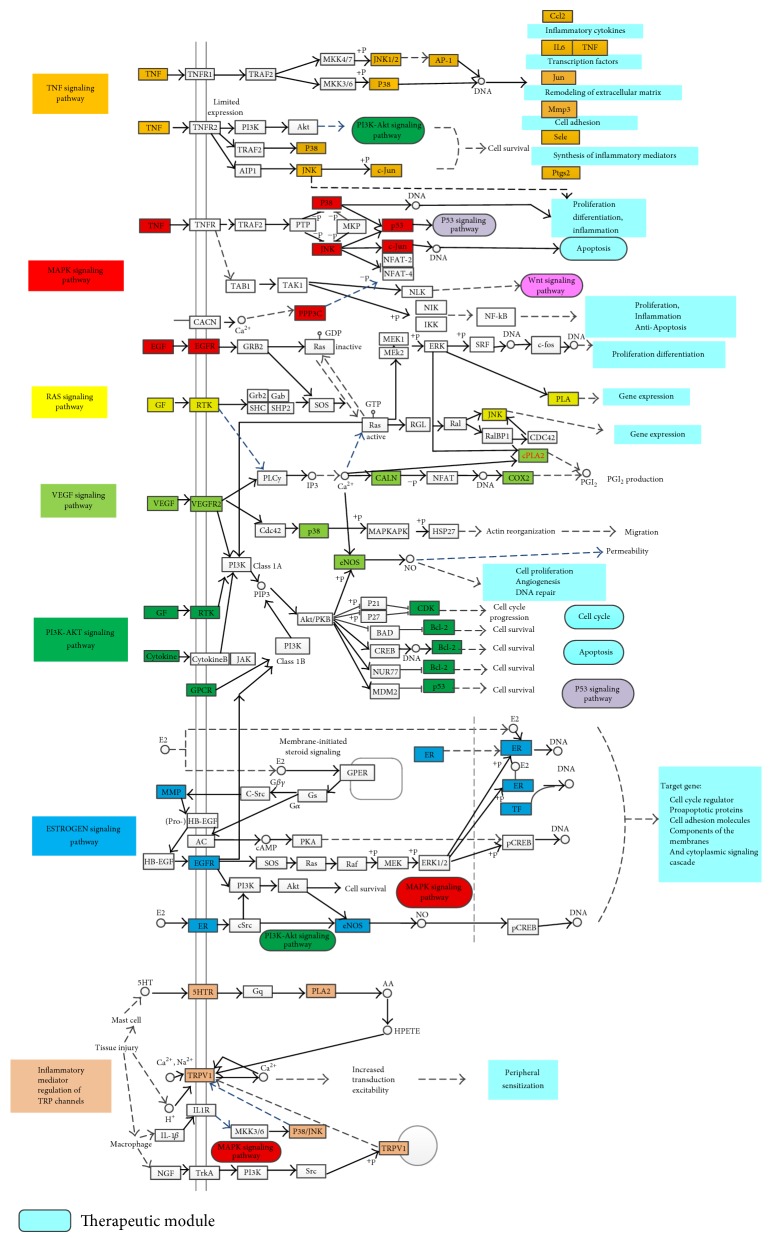
HMG pathway and therapeutic modules. The distribution of the targets on the compressed HMG pathway. Seven pathways (indicated in different colors) constitute the compressed HMG pathway. The solid and dashed arrows indicate direct and indirect activation, respectively, and the T arrows represent the inhibition effects.

To more accurately determine the molecular mechanisms of DLCs, an integrated HMG pathway was assembled by integrating the key pathways obtained from the target-pathway network analysis, including the TNF signaling pathway (ssc04668), MAPK signaling pathway (ssc04010), Ras signaling pathway (ssc04014), VEGF signaling pathway (ssc04370), phosphatidylinositol 3-kinase- (PI3K-) Akt signaling pathway (ssc04151), estrogen signaling pathway (ssc04915), and inflammatory mediator regulation of transient receptor potential (TRP) channels (ssc04750).  Figure 4 presents detailed information on the three representative therapeutic modules to clarify the molecular mechanisms of DLCs (inflammatory module, analgesic mechanism module, and hormonal regulation module).

#### 3.6.1. Inflammatory Module

The tumor necrosis factor (TNF) and mitogen-activated protein kinase (MAPK) signaling pathways are major inflammatory signaling pathways. As shown in Figure 4, TNF induces systemic inflammation by binding to two receptors (TNFR1 and TNFR2). Nevertheless, MAPKs are a family of serine-threonine-protein kinases that mediate fundamental biological processes and cellular responses to external stress signals [20]. Inflammatory responses can be inhibited by suppressing the phosphorylation of p38 in MAPK signaling pathways. DLCs may exert antihyperplasia effects through anti-inflammatory responses [21].

#### 3.6.2. Analgesic Mechanism Module

The TRP channel pathway can be indirectly modulated by inflammatory mediators. As shown in Figure 4, TRPV1 is a nonselective positive ion channel that is mainly expressed in sensory neurons and is a member of the TRP family [22, 23] and functions as a heat-sensitive, nonselective cation channel with Ca2+, Na+, and H+ [24]. Moreover, TRPV1 can be activated and sensitized by anti-inflammatory agents, such as nerve growth factor and interleukin-1*β*. TRPV1 can be activated by mechanical irritation, chemical irritation, or endogenous ligands to mediate pain and impair body functions. TRPV1 shows desensitization in a Ca^2+^-dependent manner upon repeated activation by capsaicin or protons [25]. Therefore, DLCs can exert analgesic effects through anti-inflammation.

#### 3.6.3. Hormonal Regulation Module

Estrogen is steroid hormone that can regulate many physiological processes in mammals, including reproduction and cellular homeostasis. HMG can be inhibited by adjusting the estrogen signaling pathway [26]. In the estrogen signaling pathway, estrogen mediates its cellular actions by binding to ESR1 or ESR2 and activating the MAPK and PI3K-Akt signaling pathways (Figure 4) [27]. Estrogen mediates its cellular actions through nuclear- and membrane-initiated steroid signaling by binding to either ESR1 or ESR2. These receptors belong to the nuclear receptor superfamily. A family of ligand-regulated transcription factors and ESR1-ESR2 balance are essential to maintain health [28].

## 4. Discussion

A TCM formula is composed of multiple compounds and has a complex mechanism of action, which may be associated with multiple targets and pathways in humans. DLCs are a novel drug commonly used for treating HMG and are approved by the China Food and Drug Administration. In the present study, a systems pharmacology approach was applied to identify the bioactive compounds and significant pathways of DLCs by evaluating OB and DL. We used the TCMSP database to retrieve 601 compounds present in DLCs. The results revealed favorable OB and DL properties of 77 compounds. Screening revealed that 39 compounds were active molecules in DLCs, and these compounds were selected as candidates for further analysis. On the basis of the potential targets of the 39 compounds, we obtained 10 significant pathways associated with 34 targets.

A TCM formula is a multicomponent and multitarget synergistic system that accounts for the complexity of a mixture of herbal components acting on multiple targets and diseases. Therefore, these ingredients with distinct effects and targets can act on the various aspects of a disease through multiple systems, and they interact to produce synergistic effects [29, 30]. Systems pharmacology can predict the target profiles and pharmacological actions of herbal compounds. In our study, network construction approaches were used to identify the bioactive compounds in DLCs and their potential targets and to determine the underlying mechanisms of DLCs in HMG. The integrated HMG pathway analysis in our study revealed that DLCs might simultaneously regulate multiple targets coupled with various therapeutic modules, namely, anti-inflammation, analgesia, and hormone regulation. Inflammation is a critical characteristic of many human diseases, including inflammatory and autoimmune disorders, neurodegenerative conditions, infections, cardiovascular diseases, and cancer [20]. The MAPK and TNF signaling pathways are anti-inflammatory pathways, with MAPK14 and TNF as the pivotal targets, respectively [31]. TCM formulae can exert antihyperplasia effects by alleviating inflammatory responses [21]. TCM teaches that stagnation leads to pain, and thus signs and symptoms of pain can be explained by the mechanism of stagnation. TCM exerts analgesic effects through anti-inflammation because inflammation means stagnation. The chemical compounds in DLCs can exert analgesic effects through anti-inflammation, with TRPV1 as the target. DLCs can also significantly regulate the sex hormone disorder of HMG. The underlying mechanism may be associated with the downregulation of ESR1 expression and upregulation of ESR2 expression [32]. The complex interactions between ESR1 and ESR2 are comprehensively explained by the Yin–Yang theory of TCM with the estrogen signaling pathway [33].

This study reports multicomponent therapeutics by DLCs to provide multitarget, multipathway regulation of HMG activity. The limitation of our study is that only six main herbs were analyzed, and no active components were identified in CC and CG. In future we will conduct experimental verification of the potential compounds to validate these research results based on theoretical predictions.

## 5. Conclusion

The mechanism of action of DLCs in HMG involves multiple compounds, targets, and pathways. The therapeutic effects of DLCs in HMG may be dependent on the regulation of the proteins and pathways related to anti-inflammation, analgesia, and hormone regulation. The systems pharmacology approaches developed in our study provide an alternative strategy for the comprehensive understanding of the mechanisms of DLCs in HMG.

## Supplementary Material

Supplement 1 displays the information of 603 compounds in Danlu Capsules. Supplement 2 shows the information of the 742 target proteins. Supplement 3 presents the information of the 39 active compounds. Supplement 4 shows the information of the 30 most significantly enriched GOBP terms.

## Figures and Tables

**Table 1:** Compounds in DLCs that satisfied the criteria of OB ≥ 30% and DL ≥ 0.18.

Herbs	Total	OB ≥ 30%	DL ≥ 0.18
FC	114	69 (64.2)	19 (24.5)
CM	55	26 (28.3)	11 (17.4)
RPR	119	58 (48.9)	29 (15.3)
RC	222	142 (64.0)	15 (6.8)
TL	48	27 (56.3)	7 (14.6)
RPMP	28	11 (39.3)	8 (28.6)
CC	8	0 (0.0)	0 (0.0)
CG	9	0 (0.0)	(0.0)

**Table 2:** Information regarding HMG-related targets of DLCs.

Number	Target ID	Target name	Gene
(01)	TAR00003	Nitric-oxide synthase, inducible	NOS2
(02)	TAR00006	Prostaglandin G/H synthase 1	PTGS1
(03)	TAR00016	Muscarinic acetylcholine receptor M3	CHRM3
(04)	TAR00046	Estrogen receptor	ESR1
(05)	TAR00048	Androgen receptor	AR
(06)	TAR00070	Sodium channel protein type 5 subunit alpha	SCN5A
(07)	TAR00078	Peroxisome proliferator activated receptor gamma	PPARG
(08)	TAR00086	Apoptosis regulator Bcl-2	BCL2
(09)	TAR00094	Prostaglandin G/H synthase 2	PTGS2
(10)	TAR00095	Nitric-oxide synthase, endothelial	NOS3
(11)	TAR00139	Vascular endothelial growth factor receptor 2	KDR
(12)	TAR00153	Ornithine decarboxylase	ODC1
(13)	TAR00154	Muscarinic acetylcholine receptor M4	CHRM4
(14)	TAR00165	Acetylcholinesterase	ACHE
(15)	TAR00175	5-Hydroxytryptamine 2A receptor	HTR2A
(16)	TAR00209	Progesterone receptor	PGR
(17)	TAR00210	Muscarinic acetylcholine receptor M2	CHRM2
(18)	TAR00238	72 kDa type IV collagenase	MMP2
(19)	TAR00246	Cytosolic phospholipase A2	PLA2G4A
(20)	TAR00261	Beta-2 adrenergic receptor	ADRB2
(21)	TAR00265	Tumor necrosis factor	TNF
(22)	TAR00288	Aldose reductase	AKR1B1
(23)	TAR00298	Epidermal growth factor receptor	EGFR
(24)	TAR00299	Mu-type opioid receptor	OPRM1
(25)	TAR00306	Multidrug resistance-associated protein 1	ABCC1
(26)	TAR00307	Estrogen receptor beta	ESR2
(27)	TAR00346	Urokinase-type plasminogen activator	PLAU
(28)	TAR00349	Hepatocyte growth factor receptor	MET
(29)	TAR00351	Interleukin-6	IL6
(30)	TAR00353	Interstitial collagenase	MMP1
(31)	TAR00363	Cathepsin D	CTSD
(32)	TAR00365	Interferon gamma	IFNG
(33)	TAR00374	Fatty acid synthase	FASN
(34)	TAR00402	Mitogen-activated protein kinase 14	MAPK14
(35)	TAR00404	Transient receptor potential cation channel subfamily V member 1	TRPV1
(36)	TAR00414	Transcription factor AP-1	JUN
(37)	TAR00417	C-C motif chemokine 2	CCL2
(38)	TAR00418	Interleukin-1 beta	IL1B
(39)	TAR00427	E-selectin	SELE
(40)	TAR00428	Myeloperoxidase	MPO
(41)	TAR00436	Gap junction alpha-1 protein	GJA1
(42)	TAR00441	Stromelysin-1	MMP3
(43)	TAR00444	Heat shock protein HSP 90	Hsp90
(44)	TAR00466	Tissue factor	F3
(45)	TAR00470	NAD(P)H dehydrogenase [quinone] 1	NQO1
(46)	TAR00521	Leukotriene A-4 hydrolase	LTA4H
(47)	TAR00568	Xanthine dehydrogenase/oxidase	XDH
(48)	TAR00573	Cell division protein kinase 4	CDK4
(49)	TAR00581	Neuronal acetylcholine receptor protein, alpha-7 chain	CHRNA7
(50)	TAR00597	Superoxide dismutase [Cu-Zn]	SOD1
(51)	TAR00621	Cytochrome P450 3A4	CYP3A4
(52)	TAR00646	Cellular tumor antigen p53	TP53
(53)	TAR00647	Serine/threonine-protein kinase Chk1	CHEK1
(54)	TAR00704	Mitogen-activated protein kinase 8	MAPK8
(55)	TAR00724	Cytochrome P450 1A2	CYP1A2
(56)	TAR00733	Glutathione S-transferase P	GSTP1
(57)	TAR00734	Proepidermal growth factor	EGF
(58)	TAR00740	Vascular endothelial growth factor A	VEGFA
(59)	TAR02132	Heme oxygenase 1	HMOX1
(60)	TAR02915	Retinoblastoma-associated protein	RB1
(61)	TAR02966	Protooncogene serine/threonine-protein kinase Pim-1	PIM1
(62)	TAR03204	Aryl hydrocarbon receptor	AHR
(63)	TAR03276	Nuclear receptor coactivator 2	NCOA2
(64)	TAR03279	Nuclear receptor coactivator 1	NCOA1
(65)	TAR03971	Serine/threonine-protein phosphatase 2B catalytic subunit alpha isoform	PPP3CA
(66)	TAR03978	Interleukin-2	IL2
